# Network meta-analysis incorporating randomized controlled trials and non-randomized comparative cohort studies for assessing the safety and effectiveness of medical treatments: challenges and opportunities

**DOI:** 10.1186/s13643-015-0133-0

**Published:** 2015-11-05

**Authors:** Chris Cameron, Bruce Fireman, Brian Hutton, Tammy Clifford, Doug Coyle, George Wells, Colin R. Dormuth, Robert Platt, Sengwee Toh

**Affiliations:** School of Epidemiology, Public Health and Preventive Medicine, University of Ottawa, 451 Smyth Road, Suite RGN 3105, Ottawa, ON K1H 8 M5 Canada; Department of Population Medicine, Harvard Medical School and Harvard Pilgrim Health Care Institute, 133 Brookline Avenue, 6th Floor, Boston, MA 02215 USA; Division of Research, Kaiser Permanente Northern California, 2000 Broadway, Oakland, CA 94612 USA; Ottawa Hospital Research Institute, Center for Practice Changing Research Building, Ottawa Hospital—General Campus, PO Box 201B, Ottawa, ON K1H 8 L6 Canada; Canadian Agency for Drugs and Technologies in Health, 865 Carling Ave., Suite 600, Ottawa, ON K1S 5S8 Canada; Department of Anesthesiology, Pharmacology and Therapeutics, University of British Columbia, Vancouver, BC V6T 1Z3 Canada; Department of Epidemiology and Biostatistics, McGill University, 4060 Ste Catherine W #300, Montréal, Québec H3Z 2Z3 Canada; Evidence Synthesis Group, Cornerstone Research Group Inc., 3228 South Service Road, Burlington, ON L7N 3H8 Canada

**Keywords:** Network meta-analysis, Randomized controlled trials, Observational studies, Pharmacoepidemiology, Comparative effectiveness research, Distributed research networks

## Abstract

Network meta-analysis is increasingly used to allow comparison of multiple treatment alternatives simultaneously, some of which may not have been compared directly in primary research studies. The majority of network meta-analyses published to date have incorporated data from randomized controlled trials (RCTs) only; however, inclusion of non-randomized studies may sometimes be considered. Non-randomized studies can complement RCTs or address some of their limitations, such as short follow-up time, small sample size, highly selected population, high cost, and ethical restrictions. In this paper, we discuss the challenges and opportunities of incorporating both RCTs and non-randomized comparative cohort studies into network meta-analysis for assessing the safety and effectiveness of medical treatments. Non-randomized studies with inadequate control of biases such as confounding may threaten the validity of the entire network meta-analysis. Therefore, identification and inclusion of non-randomized studies must balance their strengths with their limitations. Inclusion of both RCTs and non-randomized studies in network meta-analysis will likely increase in the future due to the growing need to assess multiple treatments simultaneously, the availability of higher quality non-randomized data and more valid methods, and the increased use of progressive licensing and product listing agreements requiring collection of data over the life cycle of medical products. Inappropriate inclusion of non-randomized studies could perpetuate the biases that are unknown, unmeasured, or uncontrolled. However, thoughtful integration of randomized and non-randomized studies may offer opportunities to provide more timely, comprehensive, and generalizable evidence about the comparative safety and effectiveness of medical treatments.

## Background

Many medical conditions exist for which there are multiple treatment options. Meta-analysis is a widely used approach for aggregating results from multiple studies to provide more robust evidence on the safety and effectiveness of various treatments [1]. However, evidence based on pair-wise meta-analysis only considers two treatments at a time. Accordingly, new meta-analytic methods have emerged to permit simultaneous comparison of multiple treatment options across studies that compare two or more treatments. These methods are most commonly referred to as *network meta-analysis (NMA)* [2, 3].

Although earlier NMAs only included randomized controlled trials (RCTs) [4], recent NMAs have begun to consider both RCTs and non-randomized studies [5–9]. In this paper, we describe NMA involving both RCTs and non-randomized comparative cohort studies—defined as cohort studies that compare two or more treatment alternatives (which may include usual care or no treatment) using observational data. We discuss some of the promises and challenges, highlight the potential application of NMA in multi-center distributed data networks, and offer insights on opportunities for improving the application of this methodology.

### Introduction to network meta-analysis

**Fig. 1:**
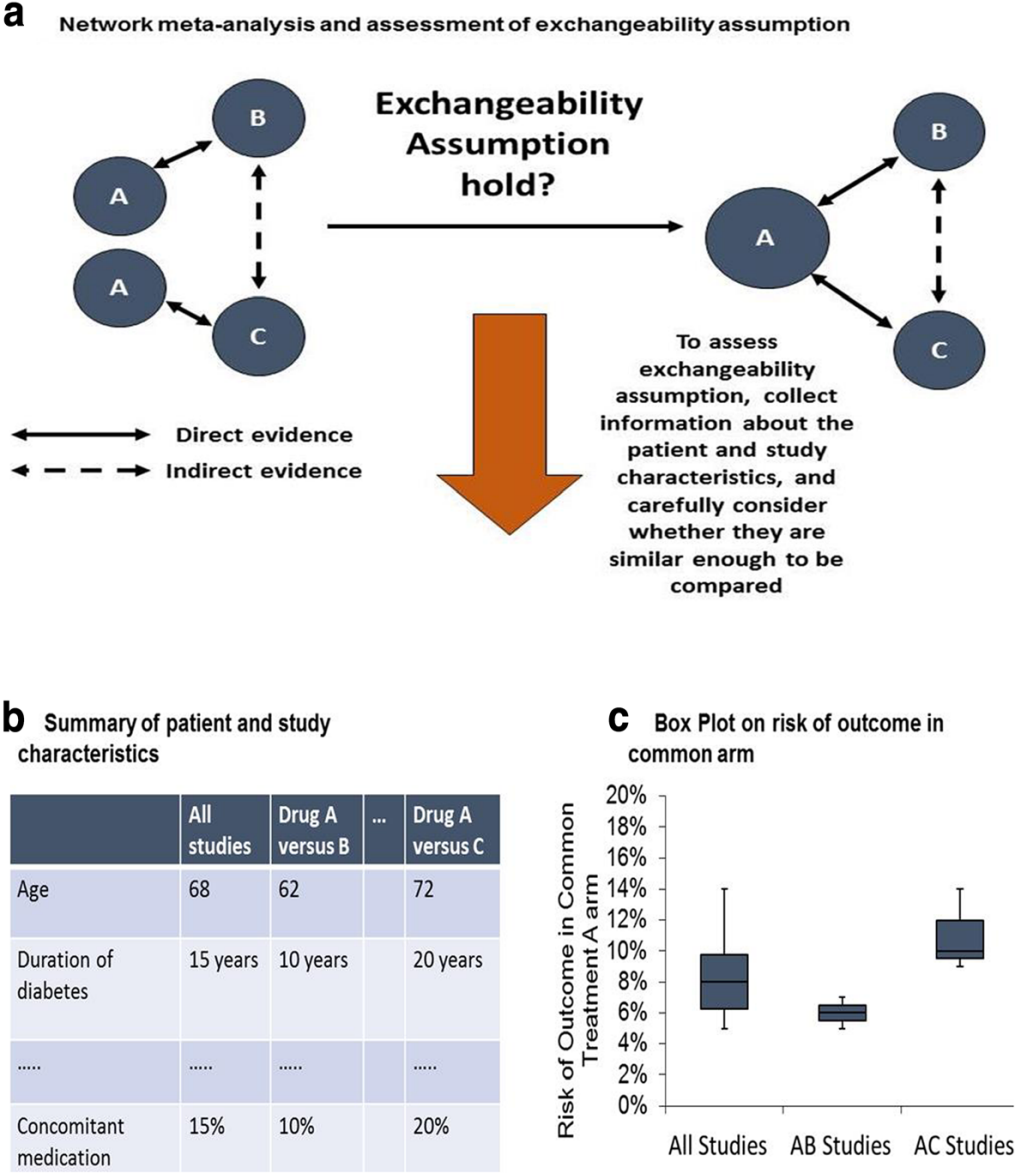
Network meta-analysis and assessment of the exchangeability assumption. Panel **a** presents a network meta-analysis assessing whether the exchangeability assumption holds for studies comparing treatments **c** versus **a** and treatments **b** versus **a**. Panel **b** presents a table comparing the patient and study characteristics for these two studies. Panel **c** assesses and plots the baseline risk of the common comparator (treatment A) for both studies and the combined result using a box plot. We have compared patient and study characteristics at the pair-wise comparison level (e.g., **a** versus **b**) although they can also be conducted at the treatment level (e.g., **a**, **b**, and **c**)

A network meta-analysis (sometimes called *mixed or multiple treatments meta-analysis*) is a method for comparing more than two interventions, some of which may not have been compared directly head-to-head in the same study (Fig. 1) [2, 3, 9–13]. The key assumption underlying any NMA is *exchangeability* of the studies [2, 3, 14]. That is, all studies measure the same underlying relative treatment effects, and any observed differences are due to chance. Stated another way, all treatments included in the NMA could have been included in the same study, and treatments are genuinely competing interventions [2, 3, 14]. For example, in Fig. 1, AC trials do not have B arms and AB trials do not have treatment C arms; however, the assumption underlying a NMA is that if an AB trial would have included a C arm, it would measure the same underlying relative effect for AC as the AC trials included in the network.

To assess exchangeability, one can collect information about the studies and carefully consider whether they appear similar enough to be compared based on inspection of this information (Fig. 1) [2, 3, 14]. Although this approach is intuitive, it can sometimes be subjective. Another way to assess exchangeability is to compare the event rate in the common treatment arm(s) [2, 3, 14]. Similar event rates may provide some reassurance that the populations are comparable. However, even if the rates differ, the exchangeability assumption may still hold if the populations do not differ in characteristics that are modifiers of the treatment effect.

Lack of exchangeability in NMA can produce discrepancy in the treatment effect estimated from direct (solid lines in panel A of Fig. 1) and indirect evidence (dashed lines in panel a of Fig. 1), sometimes also known as inconsistency [15]. There are various statistical methods to evaluate inconsistency when closed loops are available (i.e., both direct and indirect evidence are available to allow a comparison), although issues such as low statistical power may limit the applicability of some of these methods [15].

### Rationale and caveats for including non-randomized studies in NMA

With a sufficiently large sample, well-designed RCTs are expected to achieve high internal validity by balancing all measured and unmeasured prognostic factors across intervention groups through random allocation [11, 16]. However, RCTs are not without their limitations. They often have short follow-up time, small sample size, highly selected population, high cost, and ethical constraints to study certain treatments or populations. Well-designed, high-quality non-randomized studies can complement RCTs or address some of their limitations (Table 1) [17–20]. These studies may have longer follow-up time, larger sample size, and more generalizable populations who receive various treatments in real-world settings.

**Table 1:** Advantages and disadvantages of incorporating both randomized controlled trials and non-randomized comparative cohort studies in network meta-analysis

Advantages
• Non-randomized studies can complement randomized controlled trials or address some of their limitations, such as short follow-up time, small sample size, highly selected population, high cost, and ethical restrictions.
• Incorporating both types of data allows assessments of multiple treatments simultaneously, including treatments that may not have been studied in randomized controlled trials.
• Incorporating both types of data allows larger sample size and more diverse populations, thereby improving the generalizability of the findings.
• Incorporating non-randomized studies might improve network density and connect disconnected networks.
Disadvantages
• Including low-quality, non-randomized comparative cohort studies could perpetuate the biases that are unknown, unmeasured, or uncontrolled.
• There is a greater risk of violating the exchangeability assumption of network meta-analysis, especially if broad populations are considered.
• The analysis may be more complex, time- and resource-intensive, and less understood than network meta-analysis that only includes randomized controlled trials.

**Fig. 2:**
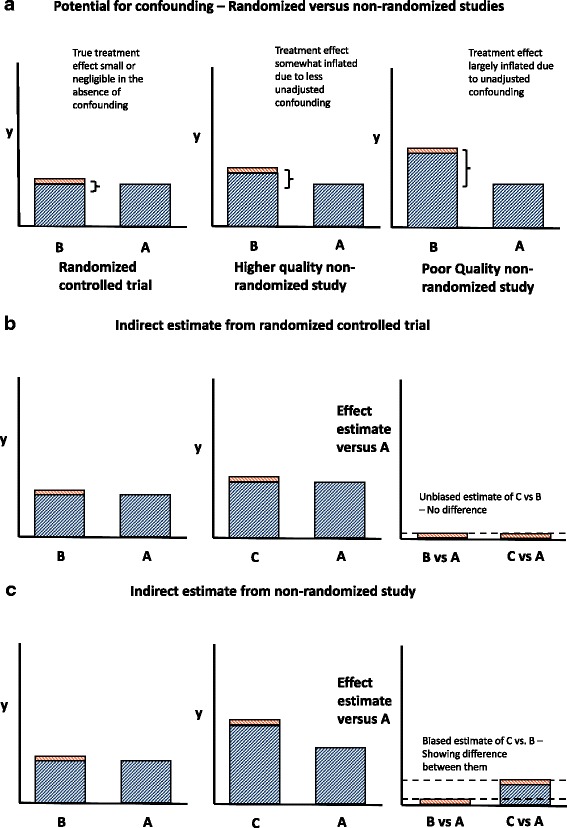
Potential bias resulting in network meta-analyses incorporating both randomized controlled trials and non-randomized comparative cohort studies. **a** Potential for confounding—randomized versus non-randomized studies. **b** Indirect estimate from randomized controlled trial. **c** Indirect estimate from non-randomized study

When considering the inclusion of both RCTs and non-randomized studies in NMA, the quality of evidence underpinning a network should be carefully assessed for each pair-wise comparison in the network. Non-randomized studies are vulnerable to several biases, including confounding which occurs when treatment groups differ in their underlying risk for the outcome [21–23]. Studies that do not appropriately account for confounding factors may therefore produce biased effect estimates (Fig. 2) [24]. Therefore, the inclusion of non-randomized studies in NMA requires careful consideration of the validity of the studies. The Grading of Recommendations Assessment, Development, and Evaluation (GRADE) working group has developed a framework for assessing the quality of evidence from non-randomized studies in the context of NMA [25]. Other guidelines, such as the STrengthening the Reporting of OBservational studies in Epidemiology (STROBE) guidelines [26] and the guidelines for good pharmacoepidemiology practices [27], also offer useful guidance to assess the quality of non-randomized studies. It is still important to carefully assess potential treatment effect modifiers even in high-quality non-randomized studies.

Another important issue to consider is whether the non-randomized studies address the same research questions or estimate the same treatment effects as the RCTs. The most commonly used analytic approach in RCTs is the intention-to-treat approach, which estimates the effect of treatment initiation. Other analyses that can be done in RCTs or non-randomized studies include as-treated analysis (which compares the treatments that the patients actually receive), per-protocol analysis (which includes only patients who adhere to the trial protocol), and other analyses such as inverse probability weighting that appropriately account for time-varying confounding [28]. Depending on analytic methods used, non-randomized studies that compare the same treatment alternatives may produce treatment effects that are valid but different from that estimated in the RCT [28–31].

### Network meta-analysis of RCTs and non-randomized studies

There are various approaches for combining RCTs and non-randomized studies in NMA [9, 13, 32, 33]. Naïve pooling of all randomized and non-randomized study-level data, using either frequentist or Bayesian NMA methods, is the simplest approach and does not differentiate between two study designs [13].

Another way to include non-randomized studies in NMA is to use them as prior information or in the form of a hierarchical model that allows for bias adjustment [13]. When incorporating them as prior information, non-randomized studies are analyzed separately and results are then used as prior information for the RCT model. The potential biases associated with non-randomized data can be modeled by adjusting the prior distribution. To downweigh the non-randomized information, the variance parameter can be inflated; to adjust for overestimation or underestimation of the treatment effect, the mean of the prior information can be shifted.

Another approach—a Bayesian hierarchical model—is generally considered the most flexible [9, 13, 32, 33]. A Bayesian hierarchical model is a statistical model that estimates the parameters of the posterior distribution using the Bayesian method [9, 13, 32, 33]. In the model, a study-design level (e.g., RCT, non-randomized study) is introduced [9, 13, 32, 33]. This approach allows for bias adjustments discussed above as well as direct comparison of study design-specific estimates to overall estimates. For example, evidence from individual studies of the same design can first be combined to produce study-design level estimates; the study-design level estimates can then be combined to obtain overall estimates [9, 13, 32, 33]. It also gives an estimate of consistency between study designs. There is limited published research in this area, especially the latter two approaches. Furthermore, there is a lack of consensus on what degree of bias adjustment to apply to non-randomized studies.

**Fig. 3:**
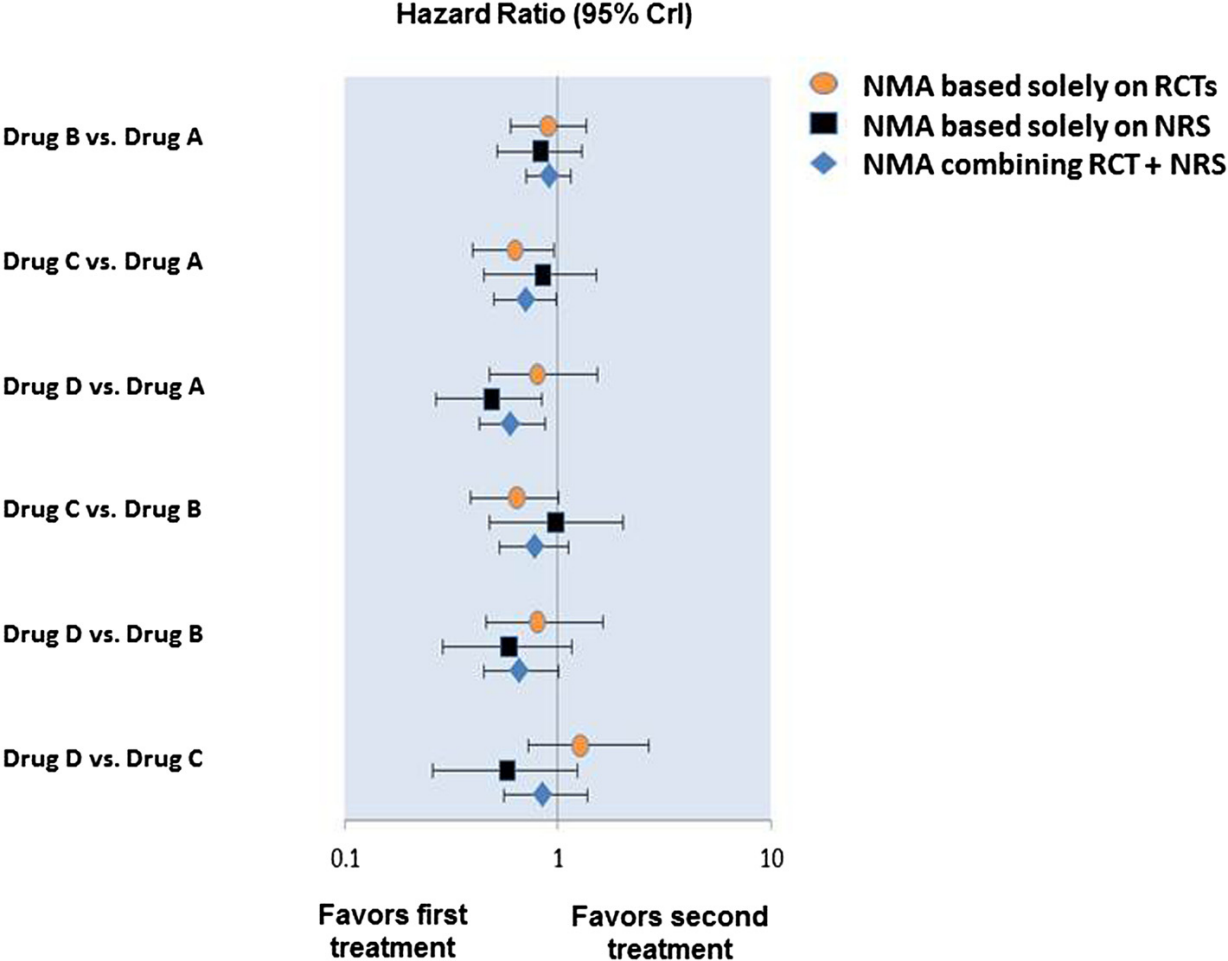
Combining and comparing findings from network meta-analysis using randomized controlled trials and non-randomized comparative cohort studies. We assume for this example a network which consists of four treatments, namely A, B, C, and D. *NMA* network meta-analysis, *NRS* non randomized studies, *RCT* randomized controlled trials

Figure 3 presents scenarios that may occur when combining RCTs and non-randomized studies in NMA. In some cases (e.g., drug B versus drug A), findings from non-randomized studies align with those reported in RCTs. In other situations (drug D versus drug C), the findings reported in the non-randomized studies do not align with those reported in RCTs. Investigators and decision makers are generally more likely to have confidence in estimates in the scenario where findings from both study designs are consistent compared with the scenario where there are discrepancies. However, the discrepancies may yield insight regarding biases in the non-randomized studies (e.g., residual confounding), effect modification by specific patient characteristic, or differences in various treatment effects (e.g., intention-to-treat effects and as-treated effects) that may not have been noticed had both study designs not been considered.

Incorporation of both RCTs and non-randomized studies into NMA typically requires considerably more time, effort, and costs compared to including only RCTs. The decision to include non-randomized studies should carefully consider the expected additional benefits given the additional time, effort, and costs. Restricting the analysis to specific types of non-randomized design or analysis (i.e., propensity score matching) may sometimes reduce time, effort, and costs to conduct NMA but may introduce bias due to exclusion of otherwise eligible studies.

### Network meta-analysis of non-randomized studies in large distributed data networks

Over the past number of years, we have seen an increase in the development of distributed data networks to assist in conducting non-randomized studies. In the USA, the Mini-Sentinel program [34] has developed a distributed network of 18 data partners with information from over 178 million individuals [35], while the Canadian Network for Observational Drug Effect Studies (CNODES) [36] includes health and prescription records of over 40 million people from eight jurisdictions in Canada and abroad. Other examples of distributed networks include the “Exploring and Understanding Adverse Drug Reactions by integrative mining of clinical records and biomedical knowledge” (EU-ADR) project in Europe [37] and the Asian Pharmacoepidemiology Network (AsPEN) [38]. These networks permit comparative safety and effectiveness assessment of medical products across multiple databases without creation of a central data warehouse [34, 36, 39].

Both pair-wise meta-analysis and NMA are well-suited for distributed data networks. Traditionally, non-randomized studies for meta-analysis are identified by systematic review of published and unpublished studies. However, these studies often include a broad array of studies with different study questions, study designs, analytic methods, and completeness of information. Combining such heterogeneous information in meta-analysis can sometimes be problematic and challenging. On the other hand, the studies performed in distributed data networks often use common protocols, data models, or both, which improves the comparability of analysis performed at each site [34, 36, 39]. Both CNODES and Mini-Sentinel have used pair-wise meta-analysis to combine data across data sources [36, 40–43]. NMA is well-suited for incorporating data from these networks when the study compares multiple treatment options, as in a Mini-Sentinel assessment of anti-hyperglycemic agents and acute myocardial infarction [44].

Further, access to data from large distributed data networks may allow more detailed assessment and adjustment for heterogeneity and inconsistency. Larger sample sizes derived from these networks will allow detailed assessment of the benefits and harms of treatments in sub-populations that may have been understudied in RCTs. Further, access to patient-level data will facilitate the conduct of meta-regression analyses to adjust for differences in characteristics between studies. This may be particularly important, because even if the estimate from a non-randomized study is unbiased, the population may differ from those studied in RCTs.

Currently, data from most distributed data networks are only available to those involved in the networks; future work is needed investigating the advantages and disadvantages of making de-identified or summary-level data from these networks more accessible for analysis by others.

## Discussion and conclusions

The interest in and need for incorporating both RCTs and non-randomized studies in NMA will likely increase in the future due to the growing need to assess multiple treatments simultaneously, improvement in the quality and validity of non-randomized data and analytic methods, and the global movement towards progressive licensing [45] and product listing agreements [46] where information on a medical product is monitored throughout its life cycle for regulatory and reimbursement purposes. Incorporating both types of data in NMA may improve precision, allow for a wider array of treatments to be considered (i.e., expand network or connect otherwise “disconnected network”), and provide real-world and more generalizable evidence on the risks and benefits of medical treatments. However, the inclusion of low-quality, non-randomized studies with inadequate control for biases may threaten the validity of the NMA findings. More studies are needed to compare the validity of different approaches that combine RCTs and non-randomized studies in NMA. Although the inclusion of both types of data in NMA poses several methodological challenges, it also offer promises to provide more timely, comprehensive, and generalizable evidence on the comparative safety and effectiveness of medical treatments.

## Abbreviations

CNODES: Canadian Network for Observational Drug Effect Studies
NMA: network meta-analysis
RCTs: randomized controlled trials
